# Towards Understanding the Factors Shaping the Composition and Function of the *Noccaea* Microbiome in Metal-Contaminated Environments

**DOI:** 10.3390/ijms26178748

**Published:** 2025-09-08

**Authors:** Marjana Regvar, Valentina Bočaj, Jure Mravlje, Teja Pelko, Matevž Likar, Paula Pongrac, Katarina Vogel-Mikuš

**Affiliations:** 1 Biotechnical Faculty, University of Ljubljana, Jamnikarjeva 101, SI-1000 Ljubljana, Slovenia; marjana.regvar@bf.uni-lj.si (M.R.); valentina.bocaj@bf.uni-lj.si (V.B.); jure.mravlje@bf.uni-lj.si (J.M.); teja.pelko@bf.uni-lj.si (T.P.); matevz.likar@bf.uni-lj.si (M.L.); paula.pongrac@ijs.si (P.P.); 2 Jozef Stefan Institute, Jamova 39, SI-1000 Ljubljana, Slovenia

**Keywords:** *Noccaea*, *Thlaspi*, plant microbiome, metal hyperaccumulation, Brassicaceae, phytoremediation

## Abstract

*Noccaea* species (formerly *Thlaspi*) are Brassicaceae plants renowned for their capacity to hyperaccumulate zinc (Zn), cadmium (Cd), and nickel (Ni), which has made them model systems in studies of metal tolerance, phytoremediation, and plant adaptation to extreme environments. While their physiological and genetic responses to metal stress are relatively well characterised, the extent to which these traits influence microbiome composition and function remains largely unexplored. These species possess compact genomes shaped by ancient whole-genome duplications and rearrangements, and such genomic traits may influence microbial recruitment through changes in secondary metabolism, elemental composition, and tissue architecture. Here, we synthesise the current findings on how genome size, metal hyperaccumulation, structural adaptations, and glucosinolate diversity affect microbial communities in *Noccaea* roots and leaves. We review evidence from bioimaging, molecular profiling, and physiological studies, highlighting interactions with bacteria and fungi adapted to metalliferous soils. At present, the leaf microbiome of *Noccaea* species remains underexplored. Analyses of root microbiome, however, reveal a consistent taxonomic core dominated by Actinobacteria and Proteobacteria among bacterial communities and Ascomycetes, predominantly Dothideomycetes and Leotiomycetes among fungi. Collectively, these findings suggest that metal-adapted microbes provide several plant-beneficial functions, including metal detoxification, nutrient cycling, growth promotion, and enhanced metal extraction in association with dark septate endophytes. By contrast, the status of mycorrhizal associations in *Noccaea* remains debated and unresolved, although evidence points to functional colonisation by selected fungal taxa. These insights indicate that multiple plant traits interact to shape microbiome assembly and activity in *Noccaea* species. Understanding these dynamics offers new perspectives on plant–microbe co-adaptation, ecological resilience, and the optimisation of microbiome-assisted strategies for sustainable phytoremediation.

## 1. Introduction

The plant microbiome—the diverse community of microorganisms associated with plant tissues—plays a crucial role in plant health, nutrient uptake, stress tolerance, and adaptation to extreme environments [1]. Recent research has revealed that microbiome composition is not random but shaped by a variety of intrinsic plant traits, including genome size, secondary metabolite production, and tissue structure [2,3]. Yet, the genetic and physiological determinants of microbiome assembly in non-model and stress-adapted plants remain poorly understood.

One such group of interest is the genus *Noccaea* (formerly part of *Thlaspi*), which includes several species capable of thriving in metal-contaminated soils and hyperaccumulating high concentrations of zinc (Zn) [4], cadmium (Cd) [5], and nickel (Ni) [4] at concentrations that are toxic to most organisms [6]. These species represent models for studying heavy metal tolerance and phytoremediation, and, at the same time, offer a unique opportunity to explore how extreme plant adaptations influence microbial colonisation in roots and leaves.

*Noccaea* belongs to the Brassicaceae, a family of one of the most diverse lineages of flowering plants, comprising 338 genera and 3709 species [7,8]. The family likely originated around 19 million years ago in the open, dry grasslands of the eastern Mediterranean [9]. Its evolutionary success has been linked to an ancient polyploidisation event and subsequent processes such as chromosome rearrangements, gene loss or silencing, genome downsizing, and diploidisation [10,11]. These events have conferred genomic plasticity and increased mutational robustness, enhancing adaptability to environmental stress [12,13]. As a result, many Brassicaceae, including *Noccaea*, can colonise harsh, nutrient-poor, and metal-polluted habitats. Based on nuclear transcriptomic markers, the family is currently divided into six major clades (A–F), with *Noccaea*, *Thlaspi*, and *Brassica* species grouped within clade B [14].

Species of *Noccaea* have small genomes, specialised root and leaf structures, and diverse glucosinolate profiles—traits that may significantly influence the structure and function of their microbiomes. For example, small genome size has been hypothesised to enhance ecological adaptability and may affect microbial recruitment by altering metabolic and signalling networks [15]. Secondary metabolites such as glucosinolates, which are prominent in Brassicaceae, have known antimicrobial properties and may act as chemical filters for microbial colonisation [16,17]. Moreover, structural features like peri-endodermal thickenings and epidermal lignification may impose spatial and biochemical constraints on microbial entry and survival [18].

Despite increasing attention to plant–microbe interactions, the *Noccaea* microbiome remains underexplored. The microbiome of leaves and stems of *N. caerulescens* is rather similar, characterised by *Actinobacteria*, *Alphaproteobacteria* and *Chloroflexi*, a bacterial community of seeds that is carried across generations despite the soil environmental changes [19]. *Chloroflexi* are a bacterial group affected by the bioavailable concentrations of Ni in the rhizosphere soil of metal-hyperaccumulating *Alyssum murale* [20]. Functional analysis of the endophytes isolated from *Nocaea goesingensis* indicated endophytes may aid in metal hyperaccumulation and improve tolerance to metal stress [21]. The latter extends to other metal hyperaccumulators and was recently confirmed for *Sedum alfredii* [22]. Bacterial populations of the phyllosphere conveying metal tolerance in *N. caerulescens* are evolutionarily adapted to high metal concentrations in plant tissues [23]. In comparison to the above-ground organs, the root endosphere of *N. caerulescens* is enriched by several rare taxa [19]. Differences in the keystone bacterial and fungal taxa were observed in the rhizosphere of *N. praecox* from non-polluted and polluted locations, with more unique taxa in the polluted locations, presumably due to increased selection pressure [24]. The roots of metal-hyperaccumulating *Noccaea brachypetala* have a lower incidence of some fungal endophytes, while its specific bacterial communities indicate an involvement in nitrogen availability and nutrition [25]. Fungal endophytes isolated from *N. caerulescens* were demonstrated to increase plant biomass, mineral nutrition and Zn uptake in this plant species [26]. Also, several dark septate fungal endophytes can boost Cd/Zn extraction of *N. caerulescens* by promoting growth and/or accumulation [27]. In addition, endophytes of *N. caerulescens* were demonstrated to improve growth and root length of *A. thaliana* [28]. However, some fundamental questions—such as the mycorrhizal status of these species—remain controversial [29]. While *Noccaea* has long been considered non-mycorrhizal, evidence suggests that under certain conditions, specific fungal symbionts, including Glomeromycota, can colonise their roots, potentially contributing to nutrient cycling and metal tolerance [30,31,32,33].

This review synthesises the current knowledge on the genomic, physiological, and ecological traits of *Noccaea* species that influence microbiome assembly and function. We discuss how genome evolution, metal hyperaccumulation, secondary metabolism, and tissue structure interact with microbial communities in the rhizosphere and phyllosphere. By highlighting emerging evidence and identifying key gaps, we aim to provide a framework for future research on plant–microbiome co-adaptation and its relevance for phytoremediation and resilience in metal-stressed environments.

## 2. Phylogenetic Relationships Within *Noccaea* (Formerly *Thlaspi*) Species

The genus *Thlaspi* L., once considered one of the largest genera in the Brassicaceae, comprises approximately 75 species primarily distributed across Eurasia [8]. However, molecular phylogenetic studies have revealed that *Thlaspi sensu lato* (*s.l.*) is polyphyletic. This hypothesis was first raised by Meyer in 1973, but gained wide acceptance only after the advent of molecular systematics. Subsequent analyses of nuclear genes and internal transcribed spacer (ITS) regions provided strong evidence for multiple evolutionary lineages, ultimately supporting the taxonomic reorganisation of *Thlaspi s.l.* into distinct monophyletic genera—*Thlaspi sensu stricto*, *Noccaea*, *Microthlaspi*, *Noccidium*, and others [14,34,35]. The analysis of 42 *Noccaea* species indicates that the generic classifications based on seed may lead to inaccurate phylogenetic conclusions [36]. In the present manuscript, the classification of *Noccaea* species according to Flora Europaea (The Euro+Med Plantbase Project, “https://ww2.bgbm.org/EuroPlusMed/query.asp”(accessed on 29 July 2025) is followed.

*Noccaea* has emerged as a genus of particular ecological and physiological interest. Al-Shehbaz (2014) [8] presented a comprehensive monograph of *Noccaea*, describing 128 species, many of which are adapted to extreme edaphic conditions [8]. Notably, the genus includes several metal hyperaccumulators [6], as discussed in Section 5.

*Noccaea caerulescens*, the most widely studied species of the genus, has become a model system for research in metal homeostasis, adaptation to metalliferous environments, and plant–microbe interactions [37,38]. Its close relative, *N. praecox*, also exhibits extreme metal tolerance and accumulation traits, especially to Cd [31,39,40]. Phylogenetic reconstruction based on ITS rDNA has shown a 99% sequence similarity between these two species, suggesting a recent divergence in the early Pleistocene, approximately 1.2 million years ago [41]. This divergence occurred in parallel with the expansion and diversification of perennial *Noccaea* species in North America from Eurasian ancestors [42].

Nuclear gene phylogenies across Brassicaceae support a pattern of nested radiation and convergent morphological evolution [14]. This evolutionary plasticity is reflected in the repeated emergence of metal tolerance traits and compact genome sizes in several Brassicaceae lineages. The genomic simplification observed in *Noccaea* may result from ancient polyploidy followed by genome downsizing, rearrangements, and diploidisation [10,11]. These processes, though complicating phylogenetic reconstruction, are also thought to have enhanced the mutational robustness and ecological adaptability of Brassicaceae [12,13].

Understanding the evolutionary history of *Noccaea* is thus essential for interpreting its extraordinary physiological traits, including metal hyperaccumulation, compact genome size, tissue-specific structural adaptations, and unique secondary metabolite profiles. These characteristics likely shape its ability to establish highly selective interactions with root- and leaf-associated microbiota. Phylogenetic insights provide a valuable framework for exploring the ecological and functional consequences of these plant traits, especially in the context of microbiome assembly, stress adaptation, and phytoremediation.

## 3. The Large Genome Size Constraint Hypothesis

Although several whole-genome duplication (WGD) events have occurred in Brassicaceae [11,43,44,45], this has not led to a proportional increase in genome size [10], primarily due to genome downsizing, to optimise nitrogen (N) and phosphate (P) costs related to nucleic acids costs and transcriptome, as well as optimisation of cell size and water use efficiency [46].

Genome size refers to the total DNA amount in the haploid nuclear genome, expressed in the number of base pairs (used in sequencing projects), or as the C-value (DNA content of the unreplicated gametic chromosome complement = 1C), with a proportional relationship of 1pg as equivalent to 978 Mbp [47,48]. Compared to other angiosperms—with more than an 8-fold range in DNA content, or 4.4-fold when excluding allotetraploids—the genomes of most Brassicaceae are relatively small [49], with genome sizes of the metal hyperaccumulators *Arabidopsis halleri* (255 Mbp) and *Noccaea caerulescens* (267 Mbp) even smaller than those of *Thlaspi arvense* (539 Mbp), *Sinapis alba* (553 Mbp), *Caulanthus heterophyllus* (686 Mbp) [49,50], and *Noccaea praecox* (253.5 Mbp) (available in NCBI under accession GCA_051546285.1). To our knowledge, sequencing confirmations on the genome sizes of other *Noccaea* species are currently not available.

The Large Genome Size Constraint Hypothesis suggests that species with small genomes are more adaptable and capable of colonising diverse environments. In contrast, large-genome species, enriched in non-coding DNA, may face limitations due to slower cell division, lower metabolic efficiency, and reduced tolerance to stress, which could make them more prone to extinction [51]. In metal-polluted environments, where physiological plasticity and efficient stress response mechanisms are critical, a small genome size may confer a particular advantage [52,53]. This may explain why metal hyperaccumulators like *Noccaea* exhibit small and stable genomes, supporting their ecological success under extreme edaphic conditions.

**Figure 1:**
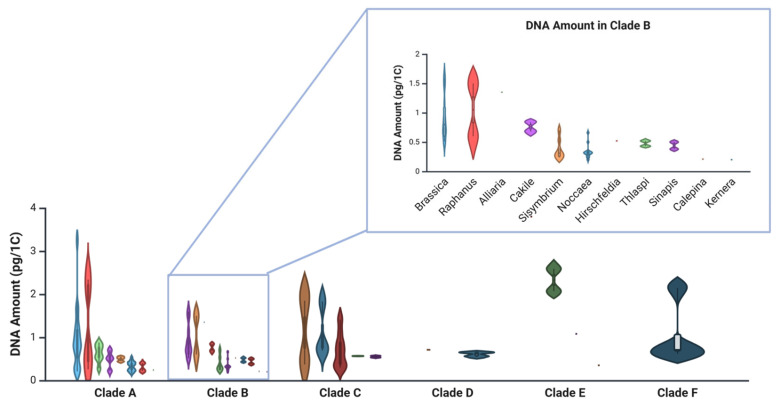
Violin plots representing the variability in the DNA amounts of the genera in the Brassicaceae clades [14], arranged according to data available at the Plant DNA C-values database [54]. Enlarged is clade B with *Noccaea* species (formerly *Thlaspi*) data corrected according to Flora Europaea (The Euro+Med Plantbase Project, https://ww2.bgbm.org/EuroPlusMed/query.asp (accessed on 29 July 2025). Dots indicate mean values, whiskers represent minimum and maximum, and kernel density outlines the distribution. Created in BioRender. Regvar, M. (2025) https://BioRender.com/jfniwhi (accessed on 4 August 2025).

According to data from the Plant DNA C-values Database [54], Brassicaceae species in clades E and F have the highest average DNA contents (1.98 pg/1C and 1.05 pg/1C, respectively) (Figure 1; Table A1), while clade B—home to *Noccaea*, *Thlaspi*, and *Brassica*—has the smallest (0.57 pg/1C) [14]. Interestingly, species retained in genus *Thlaspi* have a higher average DNA amount (0.43 pg/1C) than those assigned to *Noccaea* (0.34 pg/1C). Within *Noccaea*, *N. alpestris* and *N. praecox* have the smallest average DNA contents (0.24 and 0.26 pg/1C, respectively), indicative of extensive genome downsizing in this group. For comparison, the average DNA content in *Arabidopsis thaliana* ranges from 0.16 to 0.44 pg/1C.

## 4. Is There a Relationship Between Nuclear DNA Amount and Microbial Colonisation?

Recent metagenomic work has shown that microbial genome size is shaped by environmental context and trophic strategies [55]. Metal hyperaccumulator plants, with their chemically and structurally complex tissues, can be viewed as unique microecosystems. Hence, host genome size may also influence the structure and function of associated microbial communities.

Genome size in plants varies dramatically (0.065–152.23 pg/1C) [56,57] and correlates with traits like cell size, cell cycle control, and metabolic activity [58,59], all of which may influence microbial colonisation. As a decrease in genome size imposes a decrease in minimum cell size, species with smaller genomes acquire the potential for a greater range in cell size variation and are therefore more flexible in trait variations [60]. In line with this, the analysis of three mycotrophic plant species (from genera *Aster*, *Campanula*, *Pimpinella*) indicates a ploidy-specific response to arbuscular mycorrhizal fungi, with diploids benefiting from the interaction, while hexaploids do not [61]. In contrast, more extensive mycorrhizal colonisation was found in tetraploids compared to diploids in *Heuchera* (Saxifragaceae) [62], indicating the interaction may also be context-dependent. In addition, mycorrhizal colonisation was demonstrated to increase endopolyploidisation of root nuclei [63]. Taken together, polyploidy may alter underground interactions with beneficial microbes; however, the interaction between genome size and microbial colonisation is far from understood. Genome size may also modulate plant–microbe interactions through secondary metabolism. Expansion of specific gene families during WGD may alter secondary metabolite production and defence signalling [64], thereby affecting microbial recruitment. However, direct testing of the link between host genome size and microbial colonisation remains limited.

**Figure 2:**
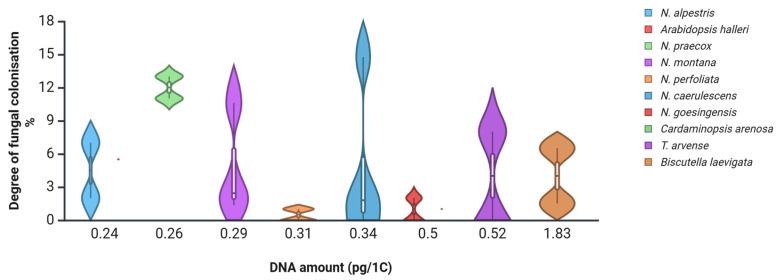
Degree of root fungal colonisation levels of several Brassicaceae species collected in polluted and non-polluted environments [66], plotted against nuclear DNA content (pg/1C) [54]. Dots indicate species averages; whiskers and kernel plots indicate distribution. Created in BioRender. Regvar, M. (2025) [31] https://BioRender.com/jfniwhi (accessed on 4 August 2025).

The ability to develop certain plant traits derives from common developmental programs and from biophysical scaling constraints that limit the minimum cell size [65]. It was recently proposed that integrating genome size, functional traits and phylogenetic data across species may shed more light on their possible relationships [60]. One approach is to estimate root fungal colonisation levels in species with known nuclear DNA amounts (1C) [54]. In *Noccaea* species collected from various polluted and non-polluted sites in Central Europe, the root fungal colonisation levels were generally low [66], supporting the hypothesis that small-genome species may restrict or tightly regulate microbial entry. When plotted against their corresponding nuclear DNA amounts (Figure 2), it seems species with medium nuclear DNA amounts (1C) may develop the highest root fungal colonisation levels. Therefore, species with extremely large and extremely small genome sizes might pose a barrier to maximal root fungal colonisation levels within the given range of phylogenetically related species. However, more evidence is needed to prove that.

**Figure 3:**
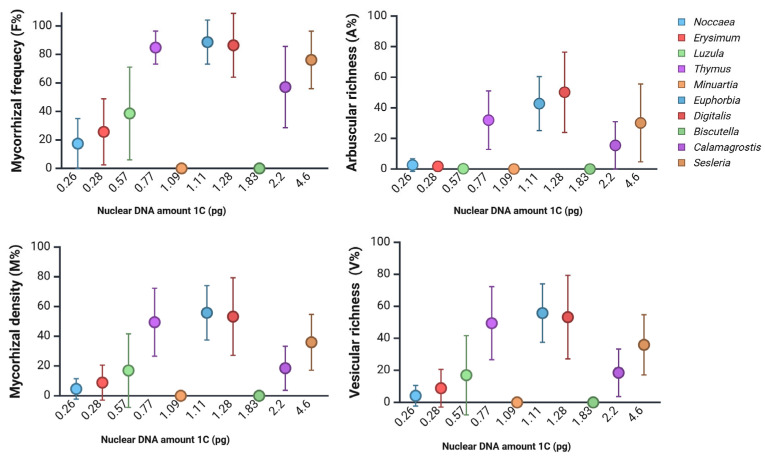
Mycorrhizal colonisation parameters of plant species from a metal-polluted site in Slovenia [32], arranged according to their corresponding DNA content (pg/1C). Data was collected in two seasons over two consecutive years (*N* = 3–20). AM was assessed according to the work of Trouvelot et al. [69]. F%, mycorrhizal frequency; M%, mycorrhizal density; A%, arbuscular richness; V%, vesicular richness. Created in BioRender. Regvar, M. (2025) [31] https://BioRender.com/92xfbu5 (accessed on 25 August 2025).

Similar findings can be obtained from data on a two-year study on vegetation at a metal-polluted site in Slovenia [32]. Within a metallophyte vegetation, plants with moderate nuclear DNA amounts tended to support higher levels of mycorrhizal colonisation compared to plants with smaller DNA amounts (Figure 3). Nevertheless, some species remained free of colonisation across all sampling periods (e.g., *Biscutella laevigata*, *Minuartia gerardii*), although *B. laevigata* collected at heavy-metal-polluted sites in Poland was reported to host arbuscular mycorrhiza (AM) [67], suggesting that factors beyond genome size—such as environmental filtering or chemical defence—may also play significant roles. To date, mycorrhizal status in Brassicaceae still remains debatable [29]. However, recent results indicate an additional pathway of beneficial effects of arbuscular mycorrhizal fungi on the growth of an invasive *Alliaria petiolata* (Brassicaceae) through an increase in soil nitrogen availability despite the lack of observable arbuscules [68].

Species identity is one of the main drivers of plant microbiome composition, followed by the presence of the metal hyperaccumulation trait [70]. The extent to which genome size impacts colonisation by beneficial microbes remains unclear, but circumstantial evidence is growing. For example, Brassicaceae-associated bacteria often have larger genomes than their free-living relatives, with enriched gene sets for secretion, carbohydrate metabolism, and stress tolerance—adaptations essential for colonising chemically complex hosts [71]. Similarly, fungal root endophytes tend to have larger genomes than foliar endophytes or saprotrophs [15], likely reflecting their need for genomic flexibility in navigating plant immunity and nutritional signals.

**Figure 4:**
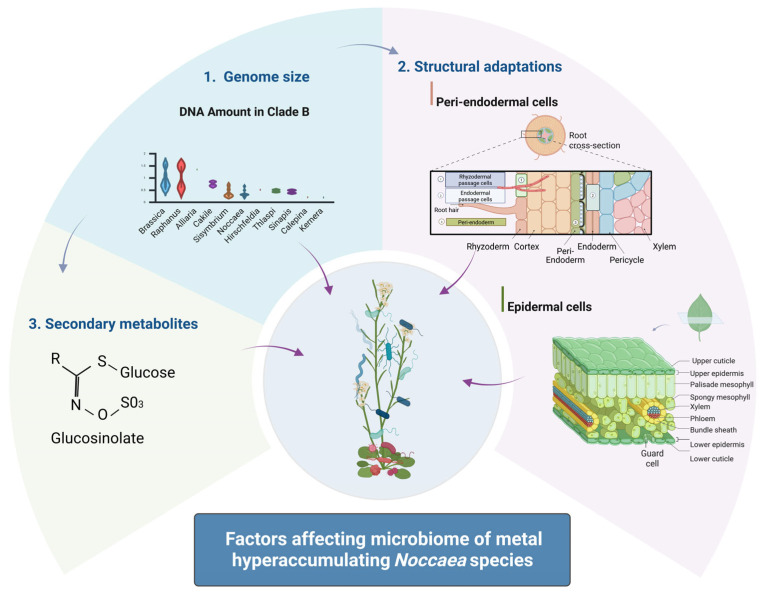
Schematic representation of the factors affecting the microbiome of metal-hyperaccumulating species. Created in BioRender. Regvar, M. (2025) https://BioRender.com/jfniwhi (accessed on 4 August 2025).

Collectively, the data supports the hypothesis that small genome size in *Noccaea* and related Brassicaceae species may confer a selective advantage under environmental stress, particularly in metal-polluted habitats. This advantage may be mediated through more efficient growth and physiological plasticity and via modulation of plant–microbe interactions (Figure 4).

While direct mechanistic links between genome size and microbiome assembly remain to be fully resolved, mounting evidence suggests that genome size influences microbial compatibility, colonisation patterns, and symbiotic potential. Future research should integrate genome-scale, metabolomic, and microbiome data to untangle these complex relationships and better understand how genome size acts as both a constraint and an opportunity in shaping plant–microbe co-evolution.

## 5. Genetic Requirements of Metal Hyperaccumulation and Impacts on the Microbiome in *Noccaea* Species

Brassicaceae hosts the greatest number of metal-hyperaccumulating species among dicotyledons, with 103 species currently listed in the Global Hyperaccumulator Database [6]. The thresholds defining hyperaccumulation are 100 mg kg^−1^ for Cd, 1000 mg kg^−1^ for Ni, and 10,000 mg kg^−1^ (1%) for zinc Zn in dry leaf tissue. According to these criteria, 23 *Noccaea* species are known to hyperaccumulate Ni, 10 species Zn, and 3 Cd [6,72].

Field-collected *N. caerulescens* populations from soils containing up to 35,790 mg kg^−1^ of Zn and 588 mg kg^−1^ of Cd were reported to accumulate 19,071 mg kg^−1^ of Zn and 164 mg kg^−1^ of Cd in their shoots [73], while several *Noccaea* species were also demonstrated to accumulate up to 30,000 mg/kg of Ni [4]. These elements are accumulated in concentrations 100–1000 times higher than in non-accumulating species [74,75].

The evolution of metal hyperaccumulation is driven by a combination of natural selection on cis-regulatory regions and gene copy number expansions [76]. In *A. halleri*, tandem triplication of the HMA4 gene (Heavy Metal ATPase 4) facilitates enhanced root-to-shoot metal transport and plays a key role in metal homeostasis, enabling colonisation of Zn- and Cd-contaminated sites [76,77]. In *N. caerulescens*, a tandem quadruplication of HMA4—arising independently from that in *A. halleri*, despite their shared ancestry over 40 Mya—suggests convergent evolution of Zn and Cd hyperaccumulation in Brassicaceae [78].

Importantly, the HMA4 copy number in *N. caerulescens* is variable among ecotypes, contributing to intraspecific differences in Cd uptake, xylem loading, and translocation [79]. Additional genomic mechanisms, such as gene inversion events enhancing expression of HMA4 and ZIP6, as well as expanded gene families related to nicotianamine biosynthesis and transport (NAS2, YSL3, ZIFL1), further contribute to the distinct metal accumulation profiles in *Noccaea* species [50].

At the physiological level, hyperaccumulation and hypertolerance involve several key processes: (i) enhanced metal uptake by roots, (ii) efficient symplastic transfer to the vascular tissue, (iii) accelerated root-to-shoot transport via the xylem, and (iv) metal sequestration in leaf vacuoles [80,81]. For example, the NcNramp1 transporter shows high expression in the roots of the Ganges ecotype, facilitating increased Cd uptake across the endodermal membrane [82]. Similarly, NcHMA4 is pivotal for Zn and Cd xylem loading and is considered a central regulator of both hyperaccumulation and tolerance [76,79]. In the shoots, vacuolar sequestration of Zn is mediated by constitutive expression of NcMTP1, encoding a Zn transporter that mitigates cytoplasmic toxicity [83]. Transcriptome analysis of the Ganges ecotype further revealed the presence of numerous isotigs encoding metal-homeostasis-related proteins, including an extra copy of Metallothionein 3 [84], a protein that binds metal ions and contributes to detoxification. Recent functional characterisation of the calcium sensor calmodulin genes suggested an important role of *NcCaM2* in metal tolerance and provided a potential target gene to enhance the metal-hyperaccumulating trait [85].

Besides plant genetics, microbial partners play an increasingly recognised role in metal accumulation and tolerance. Several fungal endophytes isolated from metalliferous sites were shown to enhance Ni uptake and biomass production in *N. caerulescens*, indicating a functional synergy between host and microbiome [86]. Comparative studies of *Noccaea* populations from metal-rich and metal-poor soils show that bacterial and fungal community composition is shaped by both edaphic factors and host genotype [24,87]. Microbial communities associated with *Noccaea* roots in metalliferous environments often harbour unique taxa with adaptations to high metal loads and show reduced antagonistic interactions, likely due to the selective pressure imposed by the toxic environment [24].

The capacity for metal hyperaccumulation in *Noccaea* species is underpinned by a complex network of genetic adaptations, including gene duplications, regulatory mutations, and physiological innovations in metal uptake, transport, and sequestration. These traits enable survival in metalliferous environments and shape the composition and function of the associated microbiome. Fungal and bacterial partners contribute to nutrient acquisition, stress mitigation, and enhanced metal tolerance, suggesting a tightly co-evolved relationship between host and microbiota. The observed intraspecific and interspecific variability in both metal-handling capacity and microbial assemblages underscores the importance of integrating genomics, ecology, and microbiome research to fully understand plant adaptation to extreme environments. Future work focusing on functional metagenomics, transcriptomics, and controlled inoculation studies will be essential to unravel the causal links between host genotype, microbial function, and environmental stress resilience in *Noccaea* and other metal-tolerant plants.

## 6. Bioimaging Techniques Reveal Adaptation Mechanisms in the Leaves of *Noccaea* Species

To withstand the “metal tsunami” posed by excessive levels of Zn, Cd, and Ni in their environment, hyperaccumulating *Noccaea* species have evolved remarkable structural and physiological adaptations (Figure 4). These adaptations are often tissue- and cell-specific and can be visualised with high-resolution bioimaging techniques. Since the tip of the leaf matures first [88], it becomes the primary site of Zn accumulation in *N. caerulescens*, whereas Ni shows a more homogeneous distribution throughout the leaf. This spatial pattern was clearly demonstrated using Laser Ablation Inductively Coupled Plasma Mass Spectrometry (LA-ICP-MS) [89], underscoring the combined influence of developmental gradients and elemental properties on the metal distribution at the organ level.

**Figure 5:**
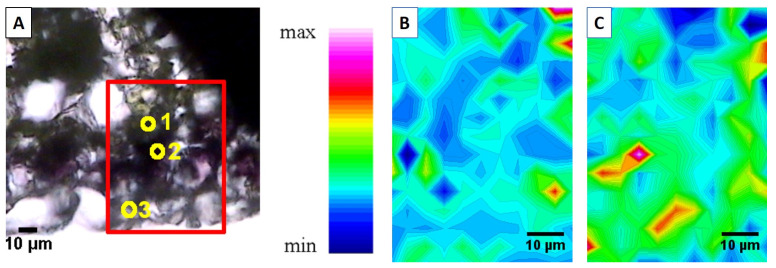
Epidermal cells ((**A**) Visible light microscopy of the selected region (x15), point 3) of field-collected *Noccaea praecox* are rich in pectin (**B**) and show lignin-reinforced cell walls (**C**), as revealed using Fourier-Transform Infrared (FTIR) chemical mapping at Synchrotron Elettra, Trieste. Points in (**A**) present (1) mesophyll, (2) sub-epidermis and (3) epidermis. (Reused from Ref. [91], licensed under CC BY 4.0).

Advanced synchrotron-based bioimaging techniques have revealed significant tissue-specific biochemical specialisation in the leaves of hyperaccumulating *Noccaea* species. For instance, Synchrotron Radiation Fourier-Transform Infrared (SR-FTIR) spectroscopy and Low-Energy X-ray Fluorescence (LEXRF) microscopy demonstrated that, besides vacuoles [90], Zn is also sequestered in the thickened cell walls of epidermal cells in *N. praecox*, where it is co-localised with lignin and pectin (Figure 5) [91]. These findings support the “egg-box” model of pectin–metal binding [92].

Meanwhile, Cd preferentially accumulates in the mesophyll at high soil Zn levels, as confirmed by micro-Particle-Induced X-ray Emission (μ-PIXE) [39,93] and in vacuoles in low Zn environment, as seen by Synchrotron micro-X-ray Fluorescence (SR-μ-XRF) [40]. These contrasting accumulation patterns suggest that mesophyll tissues serve as key sinks for Cd, while specialised epidermal compartments help to immobilise Zn, thereby limiting its cytotoxic effects.

Importantly, the spatial organisation of these metals and associated biochemical barriers, such as lignified or pectin-enriched walls, likely plays a dual role—not only in metal detoxification but also in shaping the leaf microbiome. Recent studies indicate that physical and chemical leaf traits such as pH, stomatal density, trichome presence, and even surface roughness can significantly influence microbial colonisation on leaf surfaces [94,95]. In *N. caerulescens* growing in metalliferous soils, highly specialised strains of *Pseudomonas* have been found to dominate the phyllosphere microbiome [23]. It would be of interest to explore if differences in the phyllosphere metabolome of *N. praecox* between polluted and non-polluted sites [96] are reflected in its microbiome. These findings suggest that the micro-environmental conditions created by metal compartmentalisation may act as a spatial and chemical filter, permitting colonisation only by metal-tolerant or symbiotically adapted microbes.

Even more specific are metal distributions in flowers of *N. caerulescens* and *N. praecox*, with a predominant distribution of nickel and Zn in the receptacle, ovary and anthers [97]. Interestingly, the floral microbiome of *Noccaea* species remains an enigma.

Despite compelling physiological and ecological evidence, the relationship between leaf elemental microarchitecture and microbiome spatial distribution remains unexplored. No published studies have yet integrated spatially resolved metagenomics or Fluorescence In Situ Hybridisation (FISH) with metal mapping to examine how localised chemical environments influence the microbial community structure in the phyllosphere of hyperaccumulators. Such research could provide critical insights into the co-adaptive strategies of plants and microbes under extreme environmental pressures.

Future directions should include the use of correlative imaging approaches, such as X-ray computed tomography (XCT), SR-μ-XRF, LA-ICP-MS, and confocal Raman microscopy, alongside high-resolution microbiome profiling. Integrating these tools could help uncover how micro-niches within leaves facilitate or restrict microbial colonisation, leading to a better understanding of plant–microbe–metal interactions in hyperaccumulating systems.

## 7. Specific Features in the Roots of *Noccaea* Species Affect Microbial Composition and Metal Uptake

**Figure 6:**
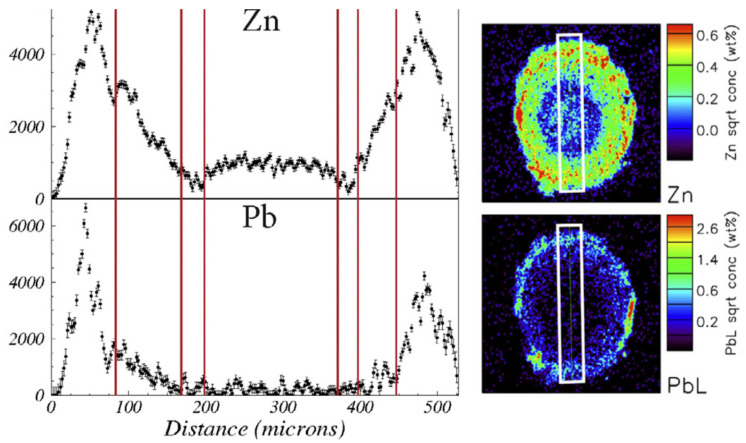
Distribution patterns of zinc and lead across the root tissues (white frame, height 500 μm) of *Viola lutea calaminaria* from a highly metal-polluted site. (reused from Ref. [99], licensed under CC BY 4.0).

The roots of *N. caerulescens* exhibit distinct anatomical specialisations, including peri-endodermal cell wall thickenings that form on the radial and inner tangential walls of cells adjacent to the endodermis (Figure 4). These structures, which resemble a “half-moon” or letter “C”, were first described by Zelko et al. 2008 [98] and later characterised using Raman spectroscopy as being rich in cellulose and lignin, with inner surfaces enriched in pectin [18]. In addition to providing mechanical support, these thickened cell walls likely function as a selective barrier, immobilising excess metals and restricting their movement toward the central vascular tissues. As a consequence, specific metal distribution patterns in root tissues are expected, similar to those observed in metallophytes from the genus *Melanium* (Figure 6), which are likely to impact microbial distribution.

Fungal entry into roots is further limited by suberin lamellae in the endodermis. However, in *A. thaliana*, it has been demonstrated that beneficial colonisation by *Colletotrichum tofieldiae* under phosphate-deficient conditions depends on the presence of unsuberised endodermal passage cells [100]. These passage cells primarily facilitate the movement of elements and signalling molecules between root layers [101,102] and may also serve as entry points for endophytic microbes. Remarkably, some evidence suggests that internalised microbes can be digested within root cells to release nitrogen that is then transported to the shoots [103].

Plant–microbe interactions in the rhizosphere are shaped by structural traits as well as by hormonal signalling. Specific bacteria can suppress abscisic acid signalling, altering the development of root diffusion barriers and indirectly influencing microbiome composition and plant stress responses [104,105]. In Brassicaceae roots, dominant fungal taxa typically belong to Ascomycota and Basidiomycota, while Glomeromycotina are generally present at a low abundance [106]. Notably, fungal communities associated with metal-hyperaccumulating *N. caerulescens* and *N. goesingensis* include members of Dothideomycetes, Sordariomycetes, Eurotiomycetes, and Leotiomycetes, many of which promote plant growth and nickel accumulation [86].

Comparative studies show that the root-associated microbiomes of *N. praecox* and *N. caerulescens*, even when collected from the same metalliferous site, exhibit no significant differences in overall structure, implying that local soil microbial communities heavily influence microbiome assembly [24]. However, *Noccaea brachypetala*, another hyperaccumulator, displays increased bacterial species richness and relative abundance of taxa associated with metal tolerance, suggesting microbial co-selection for both phytoremediation support and enhanced nutrient acquisition [83].

The mycorrhizal status of *Noccaea* species—and Brassicaceae more broadly—remains controversial, primarily because genomic analyses have revealed a consistent absence or degeneration of key genes required for AM symbiosis [29,107]. Although occasional root colonisation by AM fungi has been observed under specific conditions, these associations are typically rudimentary or facultative and often lack functional arbuscule formation. The Brassicaceae family, including *A. thaliana*, is known to lack several essential components of the canonical “symbiosis toolkit”, likely due to gene loss or pseudogenisation following ancient whole-genome duplication events [108]. Additionally, the presence of glucosinolate biosynthesis pathways—which produce antimicrobial compounds—further inhibits functional mycorrhization in this lineage. Consequently, while some *Noccaea* species may occasionally host AM fungi, e.g., during the flowering period [109,110], the majority of research supports the classification of this group as primarily non-mycorrhizal. A recent high-throughput sequencing, however, identified Glomeromycetes in the roots of *N. caerulescens* and *N. praecox*, comprising up to 9% of fungal taxa [24]. These results were supported by the successful colonisation of inoculated roots with monospore Glomeromycotina cultures and subsequent elemental shifts under controlled conditions [33,111], pointing to a context-dependent facultative mycorrhizal relationship.

In *Noccaea* species, specialised anatomical features such as peri-endodermal thickenings and endodermal suberisation influence metal sequestration, as well as microbial entry and community structure. Root-associated microbiota are shaped by intrinsic plant traits, environmental filtering in metalliferous soils, and potential symbiotic dynamics involving both fungi and bacteria. The ambiguous but emerging role of mycorrhizal fungi in *Noccaea* highlights the need for integrated approaches—combining structural imaging, metagenomics, and functional assays—to unravel the multifaceted interactions between roots, metals, and microbes. Such insights are pivotal for leveraging plant–microbe partnerships in sustainable phytoremediation and adaptation to extreme edaphic conditions.

## 8. Glucosinolates

Genome expansion in plants has been associated with alterations in secondary metabolite pathways, which can impact both microbial recruitment and defence signalling. In Brassicaceae, whole-genome duplication events followed by the silencing or loss of duplicated gene copies are believed to have facilitated gene neofunctionalisation, accelerating the evolution of glucosinolate biosynthesis genes [10,14,112]. Glucosinolates, a hallmark group of secondary metabolites in Brassicaceae, play a central role in defence against pathogens and herbivores by producing toxic isothiocyanates upon hydrolysis [113,114].

The most abundant glucosinolate in roots and leaves of *N. caerulescens* is sinalbin [115]. However, significant intraspecific variations can be found [116]. Also, glucosinolate production is developmentally specific, with the highest concentrations of sinalbin in the rosette leaves during the vegetative phase, protecting the delicate young leaves, while glucobrassicanapin peaks in roots during senescence, protecting the roots during the overwintering period, as demonstrated for *N. praecox* [110]. Interestingly, hyperaccumulation of Zn decreased sinalbin in the shoots, but not roots, of *N. caerulescens* due to reallocation of sulphur, carbon and nitrogen away from glucosinolate synthesis in the shoots, thus supporting the trade-off hypothesis between this metal and defence compounds for shoots, while the roots retain their function in defence [115].

Seasonal patterns showing increased shoot and decreased root glucosinolate levels during flowering [110,117] correspond to increased interactions with arbuscular mycorrhizal fungi [110]. In *N. praecox*, indolyl-glucosinolates dominate in the shoots, whereas roots contain approximately equal proportions of indolyl- and alkyl-glucosinolates [118]. These chemotypes may influence microbial colonisation in a tissue-specific manner. Furthermore, hyperaccumulation of Cd and Zn provides protection of *N. caerulescens* against Turnip yellow mosaic virus [119].

Recent studies indicate that glucosinolates shape microbial communities through selective pressures. For example, allyl-glucosinolates have been shown to enrich specific bacterial taxa in the phyllosphere of *A. thaliana* [120]. Moreover, the indolic glucosinolate pathway has been implicated in suppressing AM colonisation in non-host Brassicaceae [121]. In this context, the expression of transcription factors regulating aliphatic, but not indolic, glucosinolate biosynthesis in the roots of *N. caerulescens* ecotype Ganges may be a key factor in shaping its rhizosphere microbial community [84].

Beyond plant-driven biosynthesis, microbial interactions with glucosinolates further shape community structure (Figure 4). Bacterial myrosinase specificity influences glucosinolate breakdown and can affect community composition, especially when coupled with metabolic cross-feeding between microbial taxa [120]. Some fungal endophytes and soil fungi can metabolise siringin as a carbon source, although they remain sensitive to isothiocyanates [122]. Additionally, *Sclerotinia sclerotiorum* was recently found to detoxify isothiocyanates using a specific hydrolase, suggesting microbial adaptation to these potent compounds [123].

Glucosinolates in *Noccaea* species serve as potent chemical defences and as ecological gatekeepers that modulate microbial colonisation in a genotype-, tissue-, and environment-specific manner. Their biosynthesis is shaped by genome evolution and selective pressures, resulting in structurally diverse compounds with distinct effects on microbial communities. Recent studies show that specific glucosinolate classes—particularly aliphatic and indolic types—can differentially influence bacterial and fungal assemblages in roots and leaves [120,121]. However, the mechanistic pathways underlying these interactions remain largely unresolved. To fully understand how glucosinolates structure the microbiome, future research should integrate metabolomic profiling, transcriptomics, and spatially resolved microbial community analyses. Such interdisciplinary approaches will elucidate how secondary metabolites mediate plant–microbe interactions, particularly under the extreme conditions of metal-rich soils.

## 9. Conclusions and Further Directions

*Noccaea* species provide a powerful model to explore how genomic, structural, and chemical traits of plants influence microbial assembly in extreme environments such as metal-contaminated soils. Their small genome size, metal hyperaccumulation capacity, specialised leaf and root anatomy, and glucosinolate profiles act in concert to modulate root and leaf microbiomes, potentially selecting for metal-tolerant and functionally beneficial microbes. Collectively, the data supports the hypothesis that small genome size in *Noccaea* and related Brassicaceae species may confer a selective advantage under environmental stress, particularly in metal-polluted habitats. This advantage may be mediated through more efficient growth and physiological plasticity and via modulation of plant–microbe interactions (Figure 4). Although evidence supports a tight coupling between plant traits and microbial composition, the precise mechanisms—particularly the roles of spatial metal distribution, facultative mycorrhisation, and glucosinolate specificity—remain poorly understood.

Further metagenomic analyses can help to determine the structure and composition of leaves and the functional roles of the microbiota in *Noccaea*. In addition, integrative analyses of structural biochemistry (metal localisation, biomolecular composition) combined with the spatial distribution of microbiota will provide further insights into their interdependence. Furthermore, systematic analyses of phylogenetically related Brassicaceae and related metallophyte flora will shed new light on the possible relationship between microbial colonisation and genome size.

## Data Availability

Plant names were adopted according to Flora Europaea (https://ww2.bgbm.org/EuroPlusMed/query.asp). Information on DNA amount was drawn from the Plant DNA C-value database, Royal Botanical Gardens, Kew “https://ww2.bgbm.org/EuroPlusMed/query.asp” (accessed on 29 July 2025). Data on mycorrhizal colonisation is contained in the articles of Regvar et al., 2003, 2006 [32,66]. The final table is provided in Appendix A. The images were prepared by Biorender. AI was used to find references (Elicit) and for text editing (Grammarly, ChatGPT 5).

## Abbreviations

The following abbreviations are used in this manuscript:

AM	Arbuscular mycorrhiza
DNA	Deoxyribonucleic Acid
FTIR	Fourier-Transform Infrared Spectroscopy
HMA4	Heavy Metal ATPase 4
LA-ICP-MS	Laser Ablation Inductively Coupled Plasma Mass Spectrometry
LEXRF	Low-Energy X-Ray Fluorescence
NAS2	Nicotianamine Synthase 2
NcHMA4	*Noccaea caerulescens* Heavy Metal ATPase 4
NcMTP1	*Noccaea caerulescens* Metal Tolerance Protein 1
PIXE	Particle-Induced X-ray Emission
SR-μ-XRF	Synchrotron Micro X-Ray Fluorescence
YSL3	Yellow Stripe-Like 3
XCT	X-ray Computed Tomography
WDG	Whole-Genome Duplication
ZIFL1	Zinc-Induced Facilitator-Like 1
ZIP6	Cation Transporter Maintaining Zn Homeostasis

## Appendix A

### Appendix A.1

**Table A1:** Brassicaceae clades [14] with their corresponding DNA amounts, available at the Plant DNA C-values database [54]. Data corrected according to Flora Europaea (The Euro+Med Plantbase Project, “https://ww2.bgbm.org/EuroPlusMed/query.asp” (accessed on 29 July 2025).

No	Clade	Genus	Species	DNA Amount	Original Reference	Clade Average DNA Amount 1C (pg)
				1C (pg)	https://ww2.bgbm.org/EuroPlusMed/query.asp	
1	Clade A	*Capsella*	*rubella*	0.22	[124]	
2	Clade A	*Capsella*	*bursa-pastoris*	0.4	[124]	
3	Clade A	*Pachycladon*	*exilis*	0.44	[124]	
4	Clade A	*Pachycladon*	*fastigiata*	0.51	[124]	
5	Clade A	*Pachycladon*	*novae-zelandiae*	0.55	[124]	
6	Clade A	*Turritis*	*glabra*	0.24	[124]	
7	Clade A	*Arabidopsis*	*thaliana*	0.16	[125]	
8	Clade A	*Arabidopsis*	*arenosa*	0.2	[124]	
9	Clade A	*Arabidopsis*	*neglecta*	0.2	[124]	
10	Clade A	*Arabidopsis*	*halleri*	0.24	[124]	
11	Clade A	*Arabidopsis*	*korshynskyi*	0.25	[126]	
12	Clade A	*Arabidopsis*	*lyrata*	0.25	[124]	
13	Clade A	*Arabidopsis*	*cebennensis*	0.29	[124]	
14	Clade A	*Arabidopsis*	*pumila*	0.34	[127]	
15	Clade A	*Arabidopsis*	*suecica*	0.35	[124]	
16	Clade A	*Arabidopsis*	*arenosa*	0.39	[124]	
17	Clade A	*Arabidopsis*	*wallichii*	0.4	[127]	
18	Clade A	*Arabidopsis*	*neglecta*	0.4	[124]	
19	Clade A	*Arabidopsis*	*thaliana*	0.44	[128]	
20	Clade A	*Arabidopsis*	*lyrata*	0.45	[129]	
21	Clade A	*Arabidopsis*	*kamchatica*	0.52	[130]	
22	Clade A	*Physaria*	*gracilis*	0.26	[124]	
23	Clade A	*Physaria*	*ovalifolia*	0.43	[124]	
24	Clade A	*Physaria*	*arctica*	0.69	[124]	
25	Clade A	*Physaria*	*didymocarpa*	2.23	[124]	
26	Clade A	*Physaria*	*bellii*	2.34	[124]	
27	Clade A	*Erysimum*	*sylvestre*	0.28	[53]	
28	Clade A	*Erysimum*	*duriaei*	0.47	[131]	
29	Clade A	*Erysimum*	*scoparium*	0.54	[132]	
30	Clade A	*Erysimum*	*bicolor*	0.58	[132]	
31	Clade A	*Erysimum*	*goniocaulon*	0.69	[133]	
32	Clade A	*Erysimum*	*bicolor*	0.76	[124]	
33	Clade A	*Erysimum*	*cheiranthoides*	0.83	[124]	
34	Clade A	*Erysimum*	*diffusum*	0.88	[134]	
35	Clade A	*Rorippa*	*lipizensis*	0.22	[135]	
36	Clade A	*Rorippa*	*sylvestris*	0.48	[134]	
37	Clade A	*Rorippa*	*palustris*	0.54	[124]	
38	Clade A	*Rorippa*	*nasturtium-aquaticum*	0.7	[136]	
39	Clade A	*Cardamine*	*impatiens*	0.21	[49]	
40	Clade A	*Cardamine*	*hirsuta*	0.23	[49]	
41	Clade A	*Cardamine*	*amara*	0.24	[137]	
42	Clade A	*Cardamine*	*glauca*	0.28	[134]	
43	Clade A	*Cardamine*	*chelidonia*	0.36	[138]	
44	Clade A	*Cardamine*	*schinziana*	0.68	[139]	
45	Clade A	*Cardamine*	*yezoensis*	0.7	[139]	
46	Clade A	*Cardamine*	*amaraeiformis*	0.71	[139]	
47	Clade A	*Cardamine*	*valida*	0.71	[139]	
48	Clade A	*Cardamine*	*yezoensis*	0.87	[139]	
49	Clade A	*Cardamine*	*flexuosa*	0.9	[140]	
50	Clade A	*Cardamine*	*yezoensis*	0.99	[139]	
51	Clade A	*Cardamine*	*schinziana*	1	[139]	
52	Clade A	*Cardamine*	*torrentis*	1.13	[139]	
53	Clade A	*Cardamine*	*yezoensis*	1.24	[139]	
54	Clade A	*Cardamine*	*asarifolia*	1.34	[141]	
55	Clade A	*Cardamine*	*diphylla*	1.62	[142]	
56	Clade A	*Cardamine*	*pratensis*	1.70	[143]	
57	Clade A	*Cardamine*	*concatenata*	3.25	[144]	0.67
1	Clade B	*Raphanus*	*sativus*	0.6	[145]	
2	Clade B	*Raphanus*	*sativus*	1.50	[146]	
3	Clade B	*Hirschfeldia*	*incana*	0.52	[124]	
4	Clade B	*Brassica*	*hirta*	0.51	[147]	
5	Clade B	*Brassica*	*tournefortii*	0.6	[148]	
6	Clade B	*Brassica*	*campestris*	0.6	[149]	
7	Clade B	*Brassica*	*nigra*	0.8	[150]	
8	Clade B	*Brassica*	*oleracea*	0.8	[146]	
9	Clade B	*Brassica*	*rapa*	0.8	[151]	
10	Clade B	*Brassica*	*napus*	1.10	[152]	
11	Clade B	*Brassica*	*juncea*	1.50	[150]	
12	Clade B	*Brassica*	*carinata*	1.60	[150]	
13	Clade B	*Sinapis*	*arvensis*	0.38	[147]	
14	Clade B	*Sinapis*	*alba*	0.50	[153]	
15	Clade B	*Cakile*	*maritima*	0.68	[124]	
16	Clade B	*Cakile*	*edentula*	0.84	[144]	
17	Clade B	*Sisymbrium*	*officinale*	0.24	[124]	
18	Clade B	*Sisymbrium*	*loeselii*	0.24	[138]	
19	Clade B	*Sisymbrium*	*altissimum*	0.26	[138]	
20	Clade B	*Sisymbrium*	*orientale*	0.31	[49]	
21	Clade B	*Sisymbrium*	*austriacum*	0.36	[124]	
22	Clade B	*Sisymbrium*	*irio*	0.53	[49]	
23	Clade B	*Sisymbrium*	*strictissimum*	0.7	[138]	
24	Clade B	*Thlaspi*	*ceratocarpum*	0.43	[124]	
25	Clade B	*Thlaspi*	*arvense*	0.52	[124]	
26	Clade B	*Alliaria*	*petiolata*	1.35	[154]	
27	Clade B	*Calepina*	*irregularis*	0.21	[124]	
28	Clade B	*Noccaea*	*alpestris*	0.24	[124]	
29	Clade B	*Noccaea*	*alpestris*	0.2	[143]	
30	Clade B	*Noccaea*	*caerulescens*	0.34	[155]	
31	Clade B	*Noccaea*	*montana*	0.29	[155]	
32	Clade B	*Noccaea*	*goesingensis*	0.5	[156]	
33	Clade B	*Noccaea*	*oxyceras*	0.34	[157]	
34	Clade B	*Noccaea*	*praecox*	0.26	[52]	
35	Clade B	*Noccaea*	*perfoliata*	0.31	[155]	
36	Clade B	*Noccaea*	*rosularis*	0.32	[157]	
37	Clade B	*Noccaea*	*tymphaea*	0.32	[155]	
38	Clade B	*Noccaea*	*tymphaea*	0.66	[155]	
39	Clade B	*Noccaea*	*violascens*	0.31	[157]	
40	Clade B	*Kernera*	*saxatilis*	0.2	[124]	0.57
1	Clade C	*Iberis*	*sempervirens*	0.56	[124]	
2	Clade C	*Iberis*	*gibraltarica*	0.57	[124]	
3	Clade C	*Lobularia*	*libyaca*	0.53	[124]	
4	Clade C	*Lobularia*	*canariensis*	0.57	[132]	
5	Clade C	*Cochlearia*	*aucheri*	0.3	[157]	
6	Clade C	*Cochlearia*	*sempervivum*	0.33	[157]	
7	Clade C	*Cochlearia*	*pyrenaica*	0.4	[158]	
8	Clade C	*Cochlearia*	*danica*	0.7	[124]	
9	Clade C	*Cochlearia*	*officinalis*	0.75	[124]	
10	Clade C	*Cochlearia*	*tatrae*	1.04	[159]	
11	Clade C	*Cochlearia*	*borzaeana*	1.41	[159]	
12	Clade C	*Lunaria*	*rediviva*	0.37	[134]	
13	Clade C	*Lunaria*	*biennis*	1.85	[160]	
14	Clade C	*Biscutella*	*auriculata*	0.69	[124]	
15	Clade C	*Biscutella*	*didyma*	0.79	[157]	
16	Clade C	*Biscutella*	*laevigata*	1.83	[52]	0.79
1	Clade D	*Alyssum*	*saxatile*	0.65	[124]	
2	Clade D	*Alyssum*	*markgrafii*	0.54	[134]	
3	Clade D	*Alyssum*	*murale*	0.58	[156]	
4	Clade D	*Alyssum*	*saxatile*	0.65	[124]	
5	Clade D	*Berteroa*	*incana*	0.71	[124]	0.63
1	Clade E	*Parrya*	*nudicaulis*	1.08	[124]	
2	Clade E	*Chorispora*	*tenella*	0.35	[124]	
3	Clade E	*Bunias*	*erucago*	2.07	[161]	
4	Clade E	*Bunias*	*orientalis*	2.59	[161]	
5	Clade E	*Hesperis*	*matronalis*	3.8	[138]	1.98
1	Clade F	*Aethionema*	*saxatile*	0.62	[134]	
2	Clade F	*Aethionema*	*grandiflorum*	0.71	[124]	
3	Clade F	*Aethionema*	*schistosum*	0.71	[124]	
4	Clade F	*Aethionema*	*cordifolium*	2.14	[133]	1.05
